# Burden of severe illness associated with laboratory confirmed influenza in adults aged 50–64 years: A rapid review

**DOI:** 10.1111/irv.12955

**Published:** 2022-01-19

**Authors:** Dong Kyu Kim, Allison McGeer, Elizabeth Uleryk, Brenda L. Coleman

**Affiliations:** ^1^ Infectious Disease Epidemiology Research Unit Sinai Health Toronto ON Canada; ^2^ Dalla Lana School of Public Health University of Toronto Toronto ON Canada; ^3^ E.M. Uleryk Consulting Mississauga ON Canada

**Keywords:** adults, case fatality, hospitalization, influenza, mortality, surveillance

## Abstract

**Background:**

While the high burden of illness caused by seasonal influenza in children and the elderly is well recognize, less is known about the burden in adults 50–64 years of age. The lack of data for this age group is a key challenge in evaluating the cost‐effectiveness of immunization programs. We aimed to assess influenza‐associated hospitalization and mortality rates and case fatality rates for hospitalized cases among adults aged 50–64 years.

**Methods:**

This rapid review was conducted according to the PRISMA; we searched MEDLINE, EMBASE, Cochrane, Web of Science, and grey literature for articles and reports published since 2010. Studies reporting rates of hospitalization and/or mortality associated with laboratory‐confirmed influenza among adults 50–64 or 45–64 years of age for the 2010–11 through 2019–20 seasons were included.

**Results:**

Twenty studies from 13 countries were reviewed. Reported rates of hospitalization associated with laboratory‐confirmed influenza were 5.7 to 112.8 per 100,000. Rates tended to be higher in the 2015–2019 compared with the 2010–2014 seasons and were higher in studies reporting data from high‐income versus low and middle‐income countries. Mortality rates were reported in only one study, with rates ranging from 0.8 to 3.5 per 100,000 in four different seasons. The case fatality rate among those hospitalized with influenza, as reported by population‐based studies, ranged from 1.3% to 5.6%.

**Conclusions:**

Seasonal influenza imposes a significant burden of morbidity on adults 50–64 years of age but with high heterogeneity across seasons and geographic regions. Ongoing surveillance is required to improve estimates of burden to better inform influenza vaccination and other public health policies.

## 1 INTRODUCTION

Annual seasonal influenza epidemics exact considerable morbidity and mortality worldwide with an estimated 3–5 million cases of severe illness and 290,000–500,000 deaths each year.^1^ The highest burden is in young children and the elderly,^2^, ^3^, ^4^, ^5^, ^6^ and many countries' immunization recommendations emphasize vaccinating children aged 6–59 months and adults aged ≥65 years.^7^, ^8^, ^9^ Less is known about the burden of disease in adults aged 50–64 years and the vaccination coverage in this age group remains relatively low. In the 2015–2016 season, 37.9% and 43.6% of adults aged 50–64 years were vaccinated in Canada and the United States (US), respectively, compared with 64.6% and 63.4% of adults ≥65 years.^10^, ^11^ Similarly in South Korea, the influenza vaccination coverage in 2016 was 31.9% in adults aged 50–64 years and 81.7% in those aged ≥65 years.^12^

There is increasing evidence that the burden of influenza in adults aged 50–64 years is sufficient to make influenza vaccination programs cost‐effective for this age group.^13^, ^14^, ^15^, ^16^, ^17^, ^18^ For instance, in Australia, a policy to recommend and pay for influenza vaccines for all 50–64 year olds was considered likely to be cost‐beneficial for both health care payers and government, with an incremental cost‐effectiveness ratio of $22,408 per quality‐adjusted life year gained.^14^ However, these and other authors have noted that the lack of adequate epidemiological data regarding the burden of disease was a key challenge in assessing the cost‐effectiveness of influenza vaccination programs for this age group.^19^

The objective of this literature review was to describe the burden of laboratory‐confirmed influenza among adults aged 50–64 years for the 2010–2011 through 2019–2020 influenza seasons. We aimed to assess influenza‐associated hospitalization and mortality rates, as well as case‐fatality rates for hospitalized cases.

## 2 METHODS

We performed a rapid literature review following the guide by Tricco et al.^20^ and the Preferred Reporting Items for Systematic reviews and Meta‐Analyses (PRISMA).^21^, ^22^

### 2.1 Search strategy and selection criteria

A professional librarian (EU) searched MEDLINE, Medline‐in‐Process, Medline EPUBS Ahead of Print, EMBASE Classic + EMBASE (OvidSP); Cochrane (Wiley); Web of Science (Clarivate Analytics) and Scopus (ScienceDirect) databases on 13 April 2021. Grey literature sources including Microsoft Academic, science.gov, Health Canada, Open Grey, and Google Scholar were searched on 14–16 April 2021. We used database subject terms and text words for influenza, hospitalization or intensive care or ventilation or mortality, and middle‐aged adults (50 to 64 years). We limited the search to articles published since 2010. We refined and expanded search terms as required to ensure the retrieval of sentinel references. Detailed search strategies are available in section 4 of the Supporting Information.

The titles of all citations returned by the searches were scanned for relevance by one reviewer (PK). Titles were reviewed for the presence of any of the following key words: epidemiology, surveillance, burden, mortality, deaths, pneumonia, influenza‐like illness, hospitalization, or hospital admission. Articles with titles that included at least one of these words and the term influenza were retained and the abstract was reviewed. If an abstract contained information on disease burden, it was considered relevant and the full‐text was obtained for further screening.

Articles and reports were eligible for full‐text review if:

- influenza diagnosis was laboratory‐confirmed (i.e., by testing of respiratory specimens via reverse transcriptase or real‐time polymerase chain reaction [rt‐PCR], culture, enzyme‐linked immunosorbent assay, direct fluorescent antibody, or other rapid antigen test);
- at least one of the following outcomes was assessed: incidence of influenza‐associated hospitalization, mortality, or case fatality rate in a population‐based sample; and
- data could be extracted for adults aged 50–64 or 45–64 years.

Studies were not eligible if they presented data only for the 2009–2010 influenza season or earlier, for nosocomial influenza only, or for types/subtypes of influenza other than seasonal influenza (e.g., influenza H7N9). Three types of articles with relevant data were identified. They were categorized as (a) population‐based surveillance studies including those in which the population of the catchment area was estimated; (b) ecologic studies that provided burden estimates based on excess deaths or hospitalization during periods of influenza activity; and (c) surveillance studies that applied age group‐specific influenza positivity to the rate of people hospitalized with a severe acute respiratory illness (SARI).

### 2.2 Data extraction, analysis, and reporting

Data extraction was performed by one reviewer (PK) using a template that collected details on study characteristics including: study identifier, year of publication, study design, country (ies), region(s), season(s), and demographic characteristics of participants including age range and number of participants/patients by underlying chronic disease/immune compromised (see section 3 of the Supporting Information for more details).

Outcome information included study‐specific outcome definitions, criteria for influenza testing, timing of relevant specimen collection, and cause of death. The outcome measures included were crude and adjusted rates of influenza‐associated hospitalizations and mortality as well as case fatality rates for hospitalized cases. Data were collected on the variables used to adjust estimates, number of people at risk in each exposure group and stratum, and any risk factors assessed for influenza‐associated death or hospitalization. When available, outcome data were extracted by influenza type/subtype and for overall rates. All incidence rates were reported per 100,000 persons.

One reviewer (PK) assessed each study for the risk of bias using the Risk of Bias in Non‐randomized Studies of Intervention (ROBINS‐I) tool, the preferred method for Cochrane review of nonrandomized studies.^22^, ^23^ Studies assessed as having critical biases were excluded from this review.

## 3 RESULTS

**FIGURE 1:**
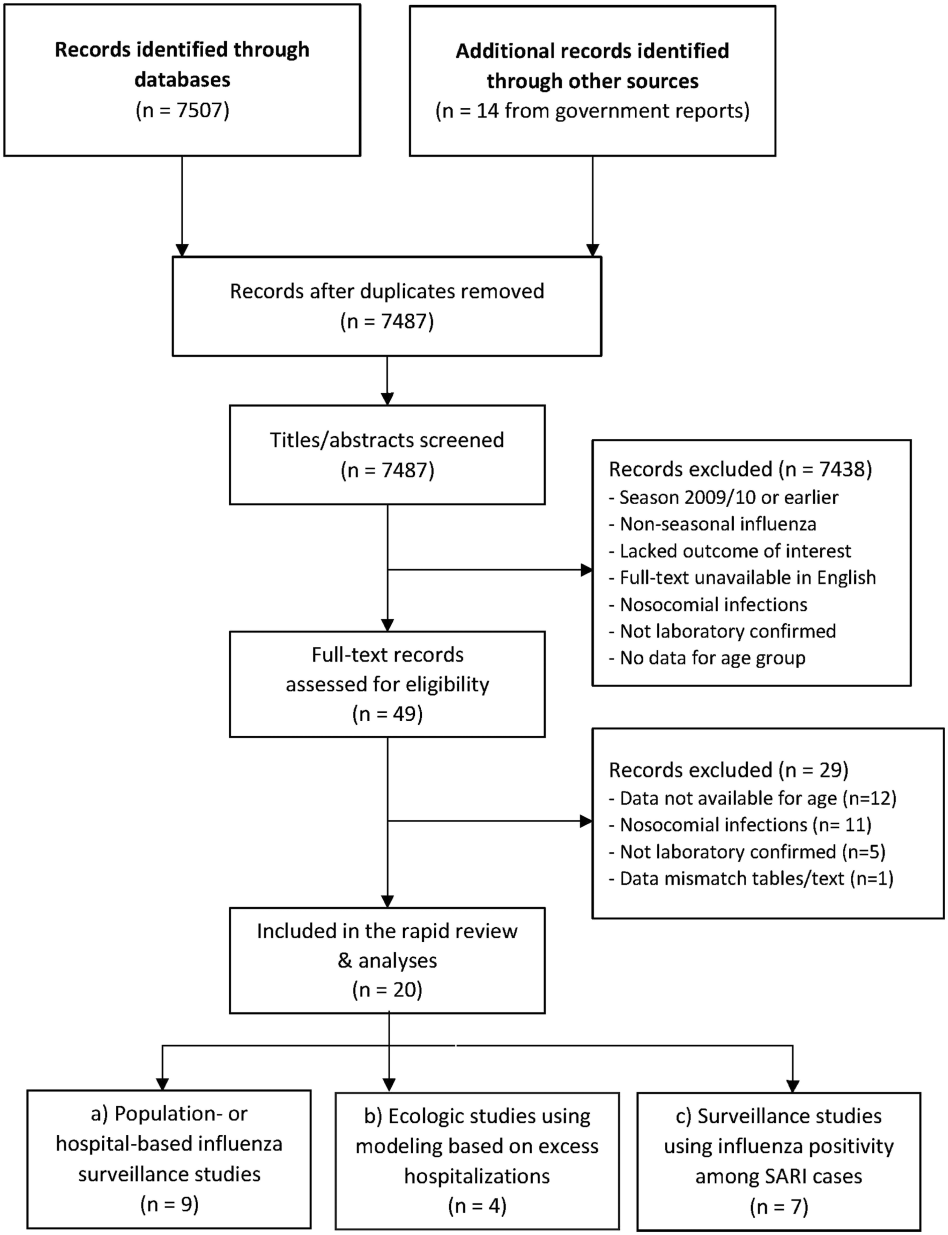
PRISMA flow diagram for literature search and study eligibility

We retrieved 7487 unique titles and abstracts of which 49 were eligible for full‐text screening (Figure 1). Overall, 20 independent primary studies^24^, ^25^, ^26^, ^27^, ^28^, ^29^, ^30^, ^31^, ^32^, ^33^, ^34^, ^35^, ^36^, ^37^, ^38^, ^39^, ^40^, ^41^ or government reports^42^, ^43^ met the inclusion criteria for review. One study^44^ was excluded because rates reported in tables and figures differed and the differences could not be resolved. Seventeen studies^24^, ^25^, ^26^, ^27^, ^31^, ^32^, ^33^, ^34^, ^35^, ^36^, ^37^, ^38^, ^39^, ^40^, ^41^, ^42^, ^43^ provided data for hospitalization, one contributed data on mortality,^35^ and four studies provided case fatality rates for hospitalized cases^28^, ^29^, ^30^, ^43^ (Table S1). All included studies were rated as moderate in overall risk of bias, and none were rejected as assessed with the ROBINS‐I tool (see Table S2).

### 3.1 Population‐based surveillance for influenza‐associated hospitalization

Six publications/reports presented data from five population‐based surveillance studies for hospitalization in the United States,^24^, ^25^, ^42^ Canada,^43^ Cambodia,^26^ and China.^27^ The three studies from the United States analysed population‐based surveillance data from the Influenza Hospitalization Surveillance Network (FluSurv‐NET) while the three studies from other countries used different measures to estimate the population in the catchment areas of participating hospitals. The rate of influenza‐associated hospitalization was available for adults aged 50–64 years (United States^24^, ^25^, ^42^ and Cambodia^26^) or 45–64 years (Canada^43^ and China^27^). Table 1 shows the hospitalization rates reported in these studies by season.

**TABLE 1:** Rate of hospitalization for adults aged 50–64 or 45–64 years with laboratory confirmed influenza, 2010–2011 to 2019–2020 influenza seasons

								Hospitalization rate (per 100,000)		
First author, year of publication	Country (Data source)	Population	Indication for testing	Included admission diagnosis	Season(s)	Number of hospital inpatients	Population size	Crude	Adjusted^a^	Dominant influenza strain
CDC, 2020^42^ and Tokars, 2018^24^	United States (FluSurv‐NET)	50–64 years	Clinician's discretion	Any	2010–11	1205	5,499,620	21.9	86.1	A(H3N2)
					2011–12	432	5,346,197	8.1	33.1	A(H3N2)
					2012–13	2210	5,430,213	40.7	151.7	A(H3N2)
					2013–14	2845	5,296,266	53.7	137.9	A(H1N1)
					2014–15	2871	5,372,656	53.4	137.2	A(H3N2)
					2015–16	2450	5,372,656	45.1	117.1	A(H1N1)
					2016–17	N/A	N/A	62.7		A(H3N2)
					2017–18	N/A	N/A	112.8		A(H3N2)
					2018–19	N/A	N/A	79.2		A(H3N2)
					2019–20	N/A	N/A	89.4		A(H3N2)
Hughes, 2019^25^	United States, Utah	50–64 years	Clinician's discretion	Any	2016–17	N/A	N/A	56	91	N/A
					2017–18	N/A	N/A	87	142	N/A
Leng, 2018^26^	Cambodia	50–64 years	SARI^b^	Not described	2015	5	30,991	16.1		A(H3N2)
					2016	28	62,290	44.9		A(H1N1) & B
Yu, 2014^27^	China	45–64 years	SARI^c^	Not described	2010–11	134	224,903	60		N/A
					2011–12	56	224,903	25		N/A
PHAC, 2020^43^	Canada (FluWatch)	45–64 years	Clinician's discretion	Symptoms compatible with influenza	2013–14	N/A	N/A	29		A(H1N1)
					2014–15	N/A	N/A	16		A(H3N2)
					2015–16	N/A	N/A	37		A(H1N1)
					2016–17	N/A	N/A	22		A(H3N2)
					2017–18	N/A	N/A	41		A(H3N2)
					2018–19	N/A	N/A	40		A(H1N1)
					2019–20	N/A	N/A	23		A(H1N1)

Abbreviations: CDC, Centre for Disease Control and Prevention; ICD‐10, International Classification of Diseases Tenth Revision; N/A, not available; PHAC, Public Health Agency of Canada; SARI, severe acute respiratory illness.

^a^ Tokars and Hughes adjusted rates for the age group‐specific percentage of hospital inpatients with respiratory disease that were tested for influenza and the sensitivity and specificity of laboratory methods used.

^b^ Fever or history of fever (≥38°C), cough or sore throat, and shortness of breath or difficulty breathing in a hospitalized person with onset of symptoms within 10 days before hospitalization.

^c^ Rectal or axillary temperature ≥37.3°C and at least one of cough, sore throat, tachypnea, difficulty breathing, abnormal breath sounds on auscultation, sputum production, hemoptysis, chest pain, or chest radiograph consistent with pneumonia.

Five of the six studies^24^, ^25^, ^27^, ^42^, ^43^ specified that rt‐PCR was used to diagnose influenza. Two studies^27^, ^43^ included patients whose specimen was collected at (or within 24 h of) hospital admission, two^24^, ^42^ included patients whose positive specimen was collected within the 14 days prior to admission, and two^25^, ^27^ did not specify the timing of influenza testing. Patients were tested for influenza as per clinician's discretion/hospital policy in all but two studies^26^, ^27^; in these studies, laboratory testing for influenza was conducted only for patients who had a SARI.

The US Center for Disease Control and Prevention's (CDC) FluSurv‐NET encompassed >70 counties in 14 states (representing about 9% of the US population).^42^ The crude hospitalization rates for laboratory‐confirmed influenza among adults aged 50–64 years ranged from 8.1 (2011–2012) to 112.8 (2017–2018) per 100,000, with an overall average of 56.7 per 100,000 for the 2010–2011 to 2019–2020 seasons. Adjustment for the percentage of hospital admissions that were tested for influenza and the sensitivity and specificity of laboratory tests resulted in substantially increased estimated rates (see Table 1). Hughes et al.^25^ reported rates of hospitalization specific to the state of Utah during the 2016–2017 and 2017–2018 seasons providing both crude and similarly adjusted estimates.

The Public Health Agency of Canada (PHAC)'s FluWatch surveillance system reported the annual rate of influenza hospitalizations for adults 45–64 years of age. From 2013–2014 through 2019–2020, the rates ranged from 16 (2014–2015) to 41 (2017–2018) per 100,000.^43^ The overall average was 29.7 per 100,000.

The other two studies using population‐based surveillance limited influenza surveillance to adults hospitalized with SARI. Yu et al.^27^ conducted surveillance at four hospitals in Hubei, China. Of 442 hospital admissions for SARI in adults aged 50–64 years, 77 (17.4%) were attributable to influenza. The influenza‐associated hospitalization rate was 60 and 25 per 100,000 in 2010–2011 and 2011–2012, respectively. In Cambodia, SARI surveillance conducted at sentinel sites in three different provinces reported influenza‐associated hospitalization rates of 16.1 and 44.9 per 100,000 persons aged 50–64 years in 2015 and 2016, respectively.^26^

In a study not eligible for inclusion because the denominator was limited to cases of influenza (as opposed to being population‐based), Yokomichi et al.^45^ described the rate of influenza‐associated hospitalization among Japanese adults aged 45–64 years who had laboratory‐confirmed influenza, using health insurance claim records. During the 2012–2013 through 2015–2016 seasons, 2,872,125 influenza cases were detected and 32,771 were hospitalized resulting in a hospitalization rate of 1141 per 100,000 influenza cases.

### 3.2 Ecologic studies estimating hospitalization rates due to laboratory‐confirmed influenza

We identified four ecologic studies^31^, ^32^, ^33^, ^34^ that estimated the incidence of influenza‐attributable hospitalizations using excess hospitalizations during periods of influenza activity (Table 2A). Two studies^32^, ^34^ reported data from Singapore, the third was from the United States^31^ and the fourth was from Portugal.^33^ Three studies^32^, ^33^, ^34^ limited estimates to hospitalization for pneumonia and influenza, while the other^31^ included all‐cause hospitalizations.

**TABLE 2:** Influenza‐attributable hospitalization rates estimated in ecologic studies and SARI surveillance studies, 2010–2011 to 2019–2020 influenza seasons

First author, year of publication	Country (data source)	Population	Included diagnoses	Season(s)	Hospitalization rate (per 100,000)	Methods
**Ecologic studies**						
Goldstein, 2015^31^	United States, New York City	50–64 years	Pneumonia & influenza (ICD‐9 480‐488)	2010–11	32.1 (21.9–42.5)	Linear regression, modelled by periodic cubic splines
			All respiratory (ICD 9‐CM 460‐519)		75.6 (51.5–99.3)	
Ng, 2019^32^	Singapore	50–64 years	Pneumonia & influenza (ICD‐9 480‐487, ICD‐10 J10‐J18)	2010	55.4 (50.6–60.5)	Generalised additive negative binomial regression, cubic smoothing spline function
				2011	48.0 (42.2–53.5)	
				2012	60.7 (55.6–66.5)	
				2013	52.5 (46.2–58.6)	
				2014	56.9 (48.8–65.3)	
				2015	55.6 (49.1–61.9)	
				2016	83.2 (74.0–91.9)	
				2017	89.4 (81.1–97.9)	
Rodrigues, 2018^33^	Portugal	50–64 years	Pneumonia & influenza (ICD‐9 480‐487, ICD‐10 J10‐J18)	2010–11	21.9 (20.0–23.8)	Autoregressive integrated moving average (ARIMA) model
				2011–12	8.9 (7.4–10.4)	
				2012–13	8.7 (7.4–10.0)	
				2013–14	5.7 (4.6–6.8)	
				2014–15	14.1 (12.5–15.7)	
Ang, 2014^34^	Singapore	45–64 years	Pneumonia & influenza (ICD‐9 480‐487, ICD‐10 J10‐J18)	2010–2012	29.7 (17.9–43.5)	Negative binomial regression, modelled by natural cubic splines
**SARI surveillance studies**						
Abdel‐Hady, 2018^35^	Oman	50–64 years	ICD‐10 J09‐J18	2012	27 (20.7–33.3)	Hospital discharge + in‐hospital death × age group‐specific influenza positivity
				2013	12.1 (8.0–16.2)	
				2014	34.5 (27.7–41.3)	
				2015	62.2 (53.2–71.1)	
Dawa, 2018^36^	Kenya	50–64 years	All	2012–2014	7.3 (6.7–8.0)	Influenza positivity among patients with SARI × SARI hospitalization rates
Emukule, 2019^37^	Uganda	50–64 years	Pneumonia as diagnosed by attending clinician	2013–2016	16 (6–27)	Influenza positivity among patients with SARI × SARI hospitalization rates
Refaey, 2016^38^	Egypt	50–64 years	All	2013	89 (71–111)	Influenza positivity among patients with SARI × SARI hospitalization rates
Nyamusore, 2018^39^	Rwanda	45–64 years	All	2012–2014	12.2 (9.8–14.6)	Influenza positivity among patients with SARI × SARI hospitalization rates
Rabarison, 2019^40^	Madagascar	45–64 years	All	2011–2016	20.0 (15.1–24.9)	Influenza positivity among patients with SARI × SARI hospitalization rates
Theo, 2018^41^	Zambia	45–64 years	All	2011–2014	25.9 (18.7–33.1)	Influenza positivity among patients with SARI × SARI hospitalization rates

Abbreviations: ICD, International Classification of Diseases; SARI, severe acute respiratory illness.

In the United States, Goldstein et al.^31^ estimated the annual rate of influenza‐associated hospitalization for residents of New York City using weekly hospitalization rates for several principal diagnoses and applying incidence proxies for the major influenza subtypes. For persons aged 50–64 years, the rate of hospitalizations for pneumonia and influenza was 32.1 (95% CI 21.9, 42.5) per 100,000 in 2010–2011, while that of any respiratory diagnosis was 75.6 (95% CI 51.5, 99.3) per 100,000.

In Singapore, Ang et al.^34^ estimated the excess hospital admissions associated with influenza by using the weekly numbers of admissions for influenza and pneumonia and the weekly rate of influenza. The estimated rate of influenza‐attributable hospital admissions for 45–64 year olds was 29.7 (95% CI 17.9, 43.5) per 100,000 for 2010 through 2012. In a second study in Singapore, Ng et al.^32^ used similar methods to estimate the incidence for 50–64 year olds with reported rates ranging from 48.0 (95% CI 42.2, 53.5) to 89.4 (95% CI 81.1, 97.9) per 100,000 for 2010 through 2017.

The estimated rate of influenza‐related hospitalizations among 50–64 year olds in Portugal for 2010–2011 through 2014–2015 ranged from 5.7 (95% CI 4.6, 6.8) to 21.9 (95% CI 20.0, 23.8) per 100,000.^33^

### 3.3 SARI surveillance studies estimating hospitalization rate using influenza positivity

Seven studies estimated the rate of influenza‐attributable hospitalization by applying the age group‐specific influenza positivity among patients with SARI to the overall hospitalization rate of patients with SARI. As shown in Table 2B, there were four studies with data for 50–64 year olds (Oman,^35^ Kenya,^36^ Uganda,^37^ and Egypt^38^) and three with data for 45–64 year olds (Rwanda,^39^ Madagascar,^40^ and Zambia^41^). All studies used a similar case definition for SARI (acute onset of illness with cough and either a recorded temperature of ≥38°C or history of fever). One study^35^ limited surveillance to hospital admissions with a discharge diagnosis of ICD‐10 codes J09‐18, and one^37^ limited surveillance to hospitalizations with diagnosis with pneumonia while the other five included all hospitalizations for SARI.^36^, ^38^, ^39^, ^40^, ^41^ The incidence of influenza‐attributable hospitalizations ranged from 7.3 (95% CI 6.7, 8.0) to 89 (71, 111) per 100,000 persons aged 50–64 years and 12.2 (10.0, 19.0) to 25.9 (18.7, 33.1) per persons 45–64 years old.

### 3.4 Potential contributing factors to the variability in reported rates of hospitalizations

**FIGURE 2:**
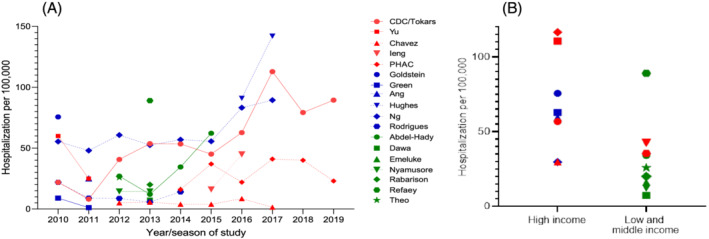
Rates of hospitalization for adults aged 50–64 years or 45–64 years with laboratory‐confirmed influenza during the 2010–2011 to 2019–2020 influenza seasons. Red, blue, and green fill denote population‐based, ecologic, and SARI surveillance studies, respectively. Shapes represent different studies and lines connect data from different years in the same study. Figure 2A shows data by season over time; for studies providing only summary data over more than one season,^34^, ^36^, ^37^, ^39^, ^40^, ^41^ data are attributed to first year/season (if data for 2 years/seasons), the 2nd if 3 or 4 years/seasons. Figure 2B shows data by study type and World Bank country income classification. The overall average rate was used for studies that provided data for more than one season^24^, ^25^, ^27^, ^32^, ^33^, ^35^, ^42^, ^43^

Data from both overall and multiyear surveillance studies for a geographical region suggested an increasing trend in rates of hospitalization associated with influenza in the more recent years of the decade included in our study (Figure 2A). The 5‐year average rate of hospitalization using population‐based surveillance in the United States for 2010–2011 through 2014–2015 was 35.3 per 100,000 compared with a rate of 77.8 per 100,000 for 2015–2016 to 2019–2020.^42^ Similar trends were reported from Canada,^43^ Singapore,^32^ and Oman.^35^

Rates reported by population‐based surveillance studies ranged from 8.1 to 112.8 per 100,000; those from ecologic studies ranged from 5.7 to 89.4 per 100,000 and those from SARI surveillance studies ranged from 7.3 to 89 per 100,000. Comparison of hospitalization rates in countries of different income levels as classified by the World Bank^46^ suggested that the incidence reported from population‐based surveillance or ecologic studies in high‐income countries were higher than those reported from SARI surveillance studies from low‐ and middle‐income countries (Figure 2B).

In a single circumstance, a population‐based study and an ecologic study assessed populations in the same country for the same season. The population‐based study^42^ of residents of 14 US states estimated the influenza‐associated hospitalization rate to be 21.9 (95% CI 20.0, 23.8) per 100,000 for 2010–2011; after adjusting the laboratory‐confirmed rate for under testing and the sensitivity and specificity of laboratory methods used, the estimate increased to 86.1 per 100,000.^24^ In comparison, the ecologic study of influenza hospitalization in New York City residents reported a rate of 32.1 (95% CI 21.9, 42.5) per 100,000 for pneumonia and influenza related hospital admissions and 75.6 (95% CI 51.5 99.3) per 100,000 for hospital admissions for any respiratory diagnosis.^31^

### 3.5 Hospitalization rate compared with adults aged ≥65 years

All studies included data for adults aged ≥65 years; the median ratio between the incidence among adults aged 50–64 and those ≥65 years was 4.3 (IQR 1.9–5.2). The ratio identified from ecologic studies (median 5.2, IQR 4.0–5.6) was significantly higher than that from population‐based (median 3.5, IQR 2.6–4.4; p = 0.05); see Figure S1.

### 3.6 Mortality and case fatality rates attributed to influenza

Among patients with SARI, the rates of influenza‐associated mortality among 50–64 year olds in Oman were 0.8 (95% CI −0.3, 1.9), 1.4 (95% CI 0, 2.7), 2.7 (95% CI 0.8, 4.5), and 3.5 (95% CI 1.4, 5.7) per 100,000 in 2012 through 2015, respectively.^35^ We identified no other studies reporting population‐based mortality rates.

The case fatality rate of patients hospitalized with laboratory‐confirmed influenza was available from four population‐based studies from the United States,^28^, ^29^ Canada,^43^ and Spain^30^ (Table 3). In the US surveillance studies, 247 of 7981 (3.1%) adults aged 50–64 years hospitalized with laboratory‐confirmed influenza died during the 2011–2012 through 2014–2015 seasons.^28^ The case fatality rate among Canadian adults aged 45–64 years from 2012–2013 through 2016–2017 was 1.3% to 5.6% by season.^43^ The case fatality rate was higher, at 11.3%, for Spanish patients with severe laboratory‐confirmed influenza, as described by Torner et al. for adults aged 45–64 years during the 2010–2011 through 2014–2015 influenza seasons.^30^

**TABLE 3:** Case fatality rate of patients with laboratory‐confirmed influenza, adults aged 50–64 years, 2010–2011 to 2019–2020 influenza seasons

First author, year of publication	Country (data source)	Population	Season(s)	Number of hospital inpatients	Number of deaths	Case‐fatality rate (%)
Arriola, 2014^28^	United States, (FluSurv‐NET)^a^	50–64 years, vaccinated	2013–14	1771	62	3.5
Collins, 2020^29^	United States, (FluSurv‐NET)^a^	50–64 years	2011–12 to 2014–15	7981	247	3.1
PHAC, 2020^43^	Canada (FluWatch, PT‐SOS)^b^	45–64 years	2012–13	843	37	4.4
			2016–17	909	37	4.1
	Canada (FluWatch, PCIRN‐SOS)^b^	45–64 years	2012–13	375	14	3.7
			2013–14	656	37	5.6
			2014–15	293	8	2.7
			2016–17	237	3	1.3
Torner, 2018^30^	Spain, Catalonia	45–64 years	2010–11 to 2014–15	382 severe cases^c^	43	11.3

Abbreviation: PHAC: Public Health Agency of Canada.

^a^ FluSurv‐NET data are collected through a network of acute care hospitals in 14 states including California, Colorado, Connecticut, Georgia, Iowa, Maryland, Michigan, Minnesota, New Mexico, New York, Ohio, Oregon, Tennessee and Utah.

^b^ Canadian data are reported by two surveillance networks: PT‐SOS (Provincial/Territorial Serious Outcomes Surveillance) that includes acute care hospital data from Newfoundland & Labrador, Prince Edward Island, Nova Scotia, New Brunswick, Manitoba, Saskatchewan, Alberta, Yukon and Northwest Territories; and CIRN‐SOS (Canadian Immunization Research Network's Serious Outcome Surveillance) that includes nine hospitals across the four provinces of Nova Scotia, Quebec, Ontario, and British Columbia.

^c^ Surveillance limited to severe cases (i.e., pneumonia, septic shock, multiorgan failure, or any other severe conditions, including ICU admissions or development of clinical signs).

### 3.7 Risk factors for death and severe outcomes

Few of the population‐based studies eligible for inclusion in this review provided data on risk factors for death in adults 50–64 years old who were hospitalized with laboratory confirmed influenza. Arriola et al.^28^ presented the risk, by influenza vaccination status, of severe outcomes of influenza. Among 1771 patients hospitalized during the 2013–2014 season, those vaccinated against influenza had a lower case fatality rate (2.2%) than those who were unvaccinated (4.2%; adjusted odds ratio: 0.48; 95% CI 0.24, 0.97). Patients who were unvaccinated also had a longer length of hospital stay (adjusted relative hazards 1.13; 95% CI 1.02, 1.26) and longer length of ICU stay (adjusted relative hazards 1.36; 95% CI 1.06, 1.74). There was no statistically significant difference in the odds of vaccinated and unvaccinated patients being admitted to ICU.

In a study comparing immunocompromised to non‐immunocompromised adults, Collins et al.^29^ reported that the crude odds of death (2.21; 95% CI 1.75, 2.78) and ICU admission (1.26; 95% CI 1.16, 1.37) were higher for immunocompromised adults in the 50–64 year old age group.

## 4 DISCUSSION

Our review identified nine population‐based surveillance, four ecologic, and seven SARI surveillance studies that reported hospitalization associated with laboratory‐confirmed influenza among adults 50–64 or 45–64 years of age, or reported population‐based estimates of case fatality rates in hospitalized persons between 2010 and 2019. The crude rate of hospitalization associated with laboratory‐confirmed influenza reported in identified studies ranged from 5.7 (Portugal, 2013–2014) to 112.8 (United States, 2017–2018) per 100,000 people 50–64 years of age.

Although data were too heterogeneous to permit pooling, the rate of hospitalization associated with laboratory‐confirmed influenza among adults aged 50–64 years (or 45–64 years) appeared to increase over time from 2010 to 2019. There was also some indication, in both population‐based surveillance and ecologic studies, that rates were lower in the 45–64 compared with the 50–64 year old age groups. In general, rates based on surveillance studies in the United States were higher than those reported from Canada. In addition, rates founded on population‐based surveillance and ecologic studies in high‐income countries were higher than those reported from any study type from low‐ and middle‐income countries.

There are multiple reasons for variability in hospitalization rates. Influenza activity varies substantially from season to season and may also vary by geographic region within one season. Other sources of variation may include differences in health care‐seeking behaviours, vaccination coverage, vaccine effectiveness, and variability in indications for and completeness of influenza testing. Studies in which surveillance of influenza was limited to patients who met a case definition for SARI^26^, ^27^ reported lower rates of hospitalization compared with those in which influenza testing was based on clinician discretion.^24^, ^25^, ^42^, ^43^ It is possible that the apparent increase in incidence over time in the United States and Canada (where influenza surveillance was based on clinician discretion) may be attributable to increased ordering of influenza testing by clinicians. The number of influenza tests has consistently increased in the United States from 2010–2011 to 2014–2015,^47^ and the studies by Tokars et al.^24^ and Hughes et al.^25^ reported that tests were not ordered for all patients that may have had influenza.

The observed disparity in rates of hospitalization for high‐income compared with low‐ and middle‐income countries may be partly attributable to a requirement for a SARI diagnosis but may also be related to differences in health care systems. It is important to note that ecologic studies of excess hospitalization during influenza seasons suggest that less than half of the total burden of influenza is directly due to pneumonia and influenza.^31^, ^32^, ^33^, ^34^ Thus, surveillance for SARI would be expected to systematically underestimate the total burden associated with influenza, as would ecologic estimates of burden considering only excess pneumonia and influenza. Consistent with this, the study by Goldstein et al. identified twice as many hospital admissions when an admission for any respiratory diagnosis was considered compared with when only pneumonia and influenza diagnoses were included.^31^ It is more difficult to assess how much of an effect this consideration may have for studies of laboratory‐confirmed influenza as both clinician discretion and hospital policies may result in substantial variation in the extent to which patients being admitted for nonrespiratory causes (e.g., myocardial infarction with fever) were tested for influenza.^48^ The significant increases in the estimated burden in US surveillance studies when cases were adjusted for under‐testing supports the argument that data in this review remain an underestimate of the true burden of influenza.^24^, ^25^

This review has limitations. We considered only articles published in peer‐reviewed English language journals or grey literature. Most of the articles we reviewed estimated, rather than measured, population denominators. Many studies were limited by the fact that influenza testing was at the discretion of the admitting practitioner while others limited their inclusion criteria to particularly severe cases or patients with SARI. Although our study eligibility criteria included only community‐acquired infections, data from the US CDC FluSurv‐NET^24^, ^42^ included nosocomial cases. However, it has been reported that hospital‐acquired influenza accounts for between 4.3% and 11.4% of total hospitalizations with laboratory‐confirmed influenza.^49^, ^50^ As such, their inclusion should not alter the estimates significantly. Also, the mortality and case fatality rates were based on analyses of in‐hospital mortality only; the potential contribution of death that occurred without hospitalization or after hospital discharge was not included, which may result in a small underestimation of deaths attributable to influenza. Finally, we were unable to control for different rates of vaccine coverage and vaccine effectiveness in the populations and seasons studied.

In sum, this review provides an updated assessment of the burden of influenza in the 50–64 year old age group that can be used to inform future studies of burden as well as the parametrization of studies of cost‐effectiveness of influenza vaccination in this age group.

## AUTHOR CONTRIBUTIONS

**Dong Kyu Kim:** Formal analysis. **Allison McGeer:** Conceptualization; funding acquisition; methodology; supervision; visualization. **Elizabeth Uleryk:** Formal analysis; methodology. **Brenda Coleman:** Conceptualization; formal analysis; methodology; project administration; supervision.

### PEER REVIEW

The peer review history for this article is available at https://publons.com/publon/10.1111/irv.12955.

## Supporting information


**Table S1:** Details of included articles on burden of influenza in adults aged 50–64 or 45–64 years
**Table S2.** Risk of Bias In Non‐randomized Studies of Intervention assessment for included studies
**Figure S1**. Median box plot of the ratio of hospitalization rates comparing 50–64 (or 45–64) year olds to ≥65 year olds, by study type

## Data Availability

Data sharing is not applicable to this article as no new data were created or analyzed in this study.
