# Supplementary material for: Burden of severe illness associated with laboratory confirmed influenza in adults aged 50–64 years: A rapid review
Source: Influenza Other Respir Viruses. 2022 Jan 19;16(4):632–42. doi: 10.1111/irv.12955 (PMC9178069; doi:10.1111/irv.12955)
Supplement: Supplementary file 1 — Table S1: Details of included articles on burden of influenza in adults aged 50–64 or 45–64 years Table S2. Risk of Bias In Non‐randomized Studies of Intervention assessment for included studies Figure S1. Median box plot of the ratio of hospitalization rates comparing 50–64 (or 45–64) year olds to ≥65 year olds, by study type [file IRV-16-632-s001.pdf]

Supplementary information

Kim *et al.* Burden of influenza in 50-64 year olds

1. **PRISMA checklist**……………………………………………………..…………...2

2. **Supplementary Tables & Figures**
   2.1 Table S1. Details of the included articles on burden of influenza in adults aged 50-64 or 45-64 years…………………………………......………………………...............4
   2.2 Table S2. Risk of Bias In Non-randomized Studies of Intervention assessment for included studies……………………………….…………………………..……...6
   2.3 Figure S1. Median box plot of the ratio of hospitalization rate between 50-64 or 45-64 year olds to ≥65 year olds across different study types…………………......……..…..7

3. **Variables Extracted from Reviewed Articles**……..…….………………….………………8

4. **Search Strategy**………………………………………………………………………11

Section 1: PRISMA CHECKLIST			
**Section and Topic**	**Item #**	**Checklist item**	**Location where item is reported**
**TITLE**			
Title	1	Identify the report as a systematic review.	Title p1 of manuscript; rapid not systematic
**ABSTRACT**			
Abstract	2	See the PRISMA 2020 for Abstracts checklist.	
**INTRODUCTION**			
Rationale	3	Describe the rationale for the review in the context of existing knowledge.	p1-2 of manuscript
Objectives	4	Provide an explicit statement of the objective(s) or question(s) the review addresses.	p2
**METHODS**			
Eligibility criteria	5	Specify the inclusion and exclusion criteria for the review and how studies were grouped for the syntheses.	p2
Information sources	6	Specify all databases, registers, websites, organisations, reference lists and other sources searched or consulted to identify studies. Specify the date when each source was last searched or consulted.	p2
Search strategy	7	Present the full search strategies for all databases, registers and websites, including any filters and limits used.	p2, Supporting Information
Selection process	8	Specify the methods used to decide whether a study met the inclusion criteria of the review, including how many reviewers screened each record and each report retrieved, whether they worked independently, and if applicable, details of automation tools used in the process.	p2-3
Data collection process	9	Specify the methods used to collect data from reports, including how many reviewers collected data from each report, whether they worked independently, any processes for obtaining or confirming data from study investigators, and if applicable, details of automation tools used in the process.	p3
Data items	10a	List and define all outcomes for which data were sought. Specify whether all results that were compatible with each outcome domain in each study were sought (e.g. for all measures, time points, analyses), and if not, the methods used to decide which results to collect.	Supporting Information
	10b	List and define all other variables for which data were sought (e.g. participant and intervention characteristics, funding sources). Describe any assumptions made about any missing or unclear information.	Supporting Information
Study risk of bias assessment	11	Specify the methods used to assess risk of bias in the included studies, including details of the tool(s) used, how many reviewers assessed each study and whether they worked independently, and if applicable, details of automation tools used in the process.	p3, Supplementary Table 2
Effect measures	12	Specify for each outcome the effect measure(s) (e.g. risk ratio, mean difference) used in the synthesis or presentation of results.	p3
Synthesis methods	13a	Describe the processes used to decide which studies were eligible for each synthesis (e.g. tabulating the study intervention characteristics and comparing against the planned groups for each synthesis (item #5)).	Supplementary Table 1
	13b	Describe any methods required to prepare the data for presentation or synthesis, such as handling of missing summary statistics, or data conversions.	N/A
	13c	Describe any methods used to tabulate or visually display results of individual studies and syntheses.	N/A
	13d	Describe any methods used to synthesize results and provide a rationale for the choice(s). If meta-analysis was performed, describe the model(s), method(s) to identify the presence and extent of statistical heterogeneity, and software package(s) used.	N/A
	13e	Describe any methods used to explore possible causes of heterogeneity among study results (e.g. subgroup analysis, meta-regression).	p10
	13f	Describe any sensitivity analyses conducted to assess robustness of the synthesized results.	N/A

Reporting bias assessment	14	Describe any methods used to assess risk of bias due to missing results in a synthesis (arising from reporting biases).	p3, Supplementary Table 1
Certainty assessment	15	Describe any methods used to assess certainty (or confidence) in the body of evidence for an outcome.	N/A
**RESULTS**			
Study selection	16a	Describe the results of the search and selection process, from the number of records identified in the search to the number of studies included in the review, ideally using a flow diagram.	Figure 1, p4
	16b	Cite studies that might appear to meet the inclusion criteria, but which were excluded, and explain why they were excluded.	p4
Study characteristics	17	Cite each included study and present its characteristics.	p4&7, Suppl Table 1
Risk of bias in studies	18	Present assessments of risk of bias for each included study.	Suppl Table 2
Results of individual studies	19	For all outcomes, present, for each study: (a) summary statistics for each group (where appropriate) and (b) an effect estimate and its precision (e.g. confidence/credible interval), ideally using structured tables or plots.	Table 1, 2, 3, Figure 2a, 2b
Results of syntheses	20a	For each synthesis, briefly summarise the characteristics and risk of bias among contributing studies.	N/A
	20b	Present results of all statistical syntheses conducted. If meta-analysis was done, present for each the summary estimate and its precision (e.g. confidence/credible interval) and measures of statistical heterogeneity. If comparing groups, describe the direction of the effect.	N/A
	20c	Present results of all investigations of possible causes of heterogeneity among study results.	p10
	20d	Present results of all sensitivity analyses conducted to assess the robustness of the synthesized results.	N/A
Reporting biases	21	Present assessments of risk of bias due to missing results (arising from reporting biases) for each synthesis assessed.	N/A
Certainty of evidence	22	Present assessments of certainty (or confidence) in the body of evidence for each outcome assessed.	N/A
**DISCUSSION**			
Discussion	23a	Provide a general interpretation of the results in the context of other evidence.	p13-14
	23b	Discuss any limitations of the evidence included in the review.	p14
	23c	Discuss any limitations of the review processes used.	p14
	23d	Discuss implications of the results for practice, policy, and future research.	p14
**OTHER INFORMATION**			
Registration and protocol	24a	Provide registration information for the review, including register name and registration number, or state that the review was not registered.	Not registered
	24b	Indicate where the review protocol can be accessed, or state that a protocol was not prepared.	Not available due to COVID-19
	24c	Describe and explain any amendments to information provided at registration or in the protocol.	N/A
Support	25	Describe sources of financial or non-financial support for the review, and the role of the funders or sponsors in the review.	p1
Competing interests	26	Declare any competing interests of review authors.	N/A
Availability of data, code and other materials	27	Report which of the following are publicly available and where they can be found: template data collection forms; data extracted from included studies; data used for all analyses; analytic code; any other materials used in the review.	Data variables collected in supporting information

Section 3: Supplementary Tables and Figures

Table S1: Details of included articles on burden of influenza in adults aged 50-64 or 45-64 years

First author, year of publication	Country (Data source)	Season(s)	Population	Included admission diagnoses	Outcome measures collected
**Population-based surveillance**					
CDC, 2020[25] and Tokars, 2018[26]	United States, (FluSurv-NET)†	2010-11 to 2019-20	50-64 years	Any	Hospitalization rate
Hughes, 2019[27]	United States, Utah	2016-17 to 2017-18	50-64 years	Any	Hospitalization rate
Ieng, 2018[28]	Cambodia	2015-16	50-64 years	Not described	Hospitalization rate
Yu, 2014[29]	China	2010-12	50-64 years	ICD-10	Hospitalization rate
PHAC, 2020[30]	Canada (FluWatch)	2014-15	45-64 years	Symptoms compatible with influenza	Hospitalization and case fatality rate
Arriola, 2017[31]	United States, (FluSurv-NET)†	2013-14	50-64 years, community dwelling	Any	Case fatality rate
Collins, 2020[32]	United States, (FluSurv-NET)†	2011-12 to 2014-15	50-64 years	Any	Case fatality rate
Torner, 2018[33]	Spain, Catalonia	2010-11 to 2014-15	45-64 years	Severe cases‡	Case fatality rate
**Ecologic studies**					
Goldstein, 2015[34]	United States, New York City	2010-11	50-64 years	Any	Hospitalization rate
Ng, 2019[35]	Singapore	2012-2017	50-64 years	Pneumonia and influenza (ICD-9 480-487, ICD-10 J10-J18)	Hospitalization rate
Rodrigues, 2018[36]	Portugal	2010-11 to 2014-15	50-64 years	Pneumonia and influenza (ICD-9 480-487, ICD-10 J10-J18)	Hospitalization rate

Ang, 2014[37]	Singapore	2010-2012	45-64 years	Pneumonia and Influenza (ICD-9 480-487, ICD-10 J10-J18)	Hospitalization rate
**Surveillance of SARI**					
Abdel-Hady, 2018[38]	Oman	2012-2015	50-64 years	ICD-10 J09-J18	Hospitalization and mortality rate
Dawa, 2018[39]	Kenya	2012-2014	50-64 years	All	Hospitalization rate
Emukule, 2019[40]	Uganda	2013-2016	50-64 years	Pneumonia as diagnosed by attending clinician	Hospitalization rate
Refaey, 2016[41]	Egypt	2013	50-64 years	All	Hospitalization rate
Nyamusore, 2018[42]	Rwanda	2012-2014	45-64 years	All	Hospitalization rate
Rabarison, 2019[43]	Madagascar	2011-2016	45-64 years	All	Hospitalization rate
Theo, 2018[44]	Zambia	2011-2014	45-64 years	All	Hospitalization rate

Abbreviations: rt-PCR: reverse transcription-polymerase chain reaction; CDC: Centre for Disease Control and Prevention; SARI: severe acute respiratory illness; ICD-10: International Classification of Diseases Tenth Revision; NP: nasopharyngeal; PHAC: Public Health Agency of Canada

[†]The Influenza Hospitalization Surveillance Network (FluSurv-NET) represents about 9% of the US population, and includes selected counties in California, Colorado, Connecticut, Georgia, Maryland, Michigan, Minnesota, New Mexico, New York, Ohio, Oregon, Tennessee, and Utah, with additional counties in Idaho (2010-11), Iowa (2012-13, 2020-21), Oklahoma (2010-11) and Rhode Island (2010-13)

Table S2. Risk of Bias In Non-randomized Studies of Intervention assessment for included studies

First authors, year of publication	Confounding	Selection bias	Classification of intervention	Intended intervention	Missing data	Measurement of outcomes	Reported results	Overall
Population-based surveillance								
CDC, 2020[25]	Moderate	Low	NI	NI	Low	Moderate	Low	Moderate
Tokars, 2018[26]	Moderate	Low	NI	NI	Low	Moderate	Low	Moderate
Hughes, 2019[27]	Moderate	Low	NI	NI	Low	Moderate	Low	Moderate
Ieng, 2018[28]	Moderate	Moderate	NI	NI	Low	Low	Low	Moderate
Yu, 2014[29]	Moderate	Moderate	NI	NI	Low	Low	Low	Moderate
PHAC, 2020[30]	Moderate	Low	NI	NI	Low	Moderate	Low	Moderate
Arriola, 2014[31]	Moderate	Low	Low	NI	Moderate	Moderate	Low	Moderate
Collins, 2020[32]	Moderate	Low	NI	NI	Low	Moderate	Low	Moderate
Torner, 2018[33]	Moderate	Moderate	NI	NI	Low	Low	Low	Moderate
Ecologic studies								
Goldstein, 2015[34]	Moderate	Low	NI	NI	Low	Moderate	Low	Moderate
Ng, 2019[35]	Moderate	Moderate	NI	NI	Low	Moderate	Low	Moderate
Rodrigues, 2018[36]	Moderate	Moderate	NI	NI	Low	Moderate	Low	Moderate
Ang, 2014[37]	Moderate	Moderate	NI	NI	Low	Moderate	Low	Moderate
SARI surveillance								
Abdel-Hady, 2018[38]	Moderate	Moderate	NI	NI	Low	Low	Low	Moderate
Dawa, 2018[39]	Moderate	Moderate	NI	NI	Low	Low	Low	Moderate
Emukule, 2019[40]	Moderate	Moderate	NI	NI	Low	Low	Low	Moderate
Refaey, 2016[41]	Moderate	Moderate	NI	NI	Low	Low	Low	Moderate
Nyamusore, 2018[42]	Moderate	Moderate	NI	NI	Low	Low	Low	Moderate
Rabarison, 2019[43]	Moderate	Moderate	NI	NI	Low	Low	Low	Moderate
Theo, 2018[44]	Moderate	Moderate	NI	NI	Low	Low	Low	Moderate

Abbreviations: NI: no information

Figure S1. Median box plot of the ratio of hospitalization rates comparing 50-64 (or 45-64) to ≥65 year olds, by study type

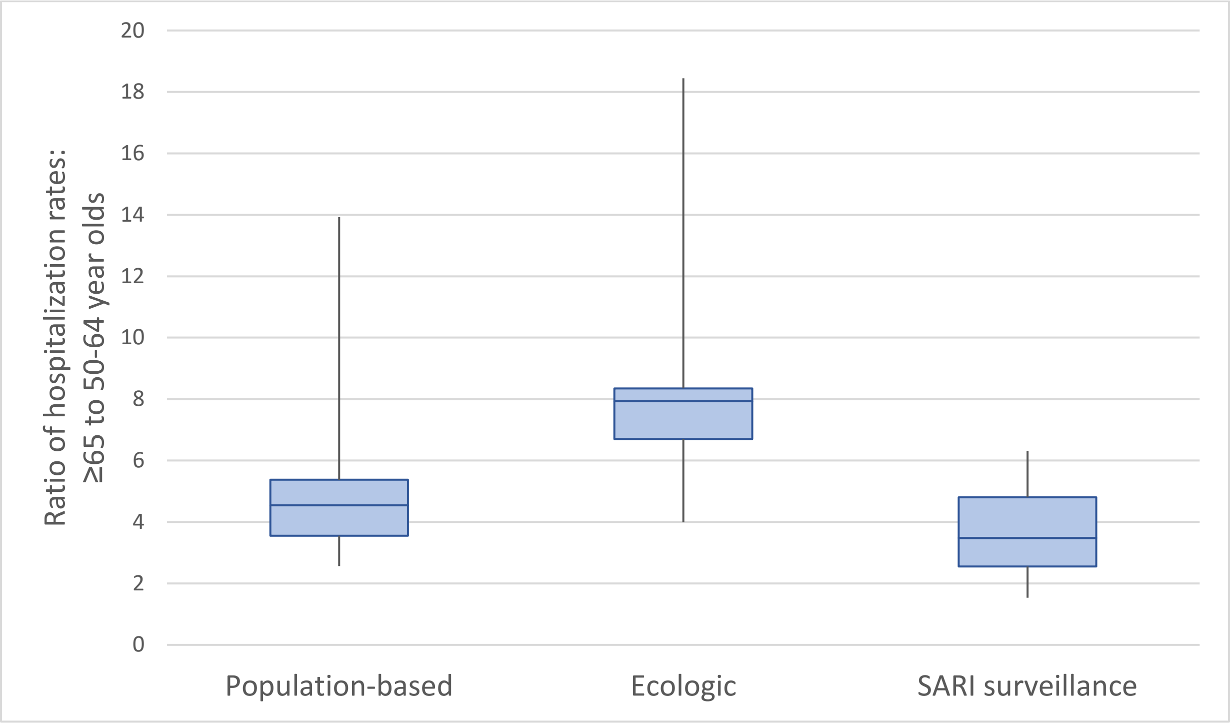

Section 3: Variables Extracted from Reviewed Articles

Study identifier

- Author's name: full name of the first author; for governmental reports, we specified the name of the government institute that published the report.
- Published year: the year of publication of the article/report.
- Country: the country in which the surveillance was conducted.
- Season: the influenza season(s) for which the surveillance was conducted.
- Study design: research study method used to measure or estimate the outcomes.
- Age range: the age groups for which the outcomes were presented; we only included articles in which data were presented for adults aged 50-64 or 45-64 years.

Outcome information

- Diagnostic testing methods used: the list of tests used to confirm laboratory diagnosis of influenza infection.
- Definition of illness leading to testing: the list of indications, signs/symptoms, hospital admission diagnoses for which laboratory testing of influenza was conducted.
- Timing of swab/testing: the window of period during which specimens were collected from patients with compatible illness(es) leading to testing to confirm influenza infection.
- Diagnosis required for inclusion: the list of hospital admission/discharge diagnoses considered in the analysis
- Definition of catchment area: the area subjected to surveillance.
- Catchment population: the population of the catchment area (i.e., number of people at risk of influenza infection)
- Number of hospital admissions: the number of adults aged 50-64 or 45-64 years hospitalized with influenza.
- Number of deaths: the number of adults aged 50-64 or 45-64 years hospitalized with influenza.
- Crude hospitalization rate (per 100,000): the rate of hospitalization associated with influenza.
- Adjusted hospitalization rate (per 100,000): the rate of hospitalization associated with influenza, with study-specific adjustments.
- Crude mortality rate (per 100,000): the rate of mortality associated with influenza.
- Adjusted mortality rate (per 100,000): the rate of mortality associated with influenza, with study-specific adjustments.
- Variables used to adjust estimate: the list of variables for which the crude hospitalization and/or mortality rates were adjusted.
- Case fatality rate: the percentage of patients who died among those hospitalized with influenza.

Section 4: Search Strategy

April 13 to 16, 2021

Database	Dates	Total	Unique	Duplicates
Cochrane	to issue 4 of 2021	368	128	240
Embase Classic + Embase	1980 to 2021 Week 14	3110	1668	1442
MEDLINE(R)	1946 to March Week 5 2021	5304	5262	42
Medline-in-Process	1946 to April 13, 2021	16	7	9
Medline Epubs Ahead of Print	to April 13, 2021	16	9	7
Hand searches				
Scopus	2010 to April 14, 2021	334	205	129
Web of Science (SCI-EXPANDED, SSCI, A&HCI, CPCI-S, CPCI-SSH, BKCI-S, BKCI-SSH, ESCI)	2010 to April 14, 2021	247	77	170
Grey literature				
Microsoft Academic	April 15, 2021	44	36	8
OpenGrey	April 15, 2021	6	6	0
Science.gov	April 15, 2021	101	36	65
CDC	April 16, 2021	1	1	0
CIC	April 16, 2021	3	3	0
ECCMID	April 16, 2021	15	14	1
ECDPC	April 16, 2021	3	3	0
Google Scholar	April 16, 2021	134	118	16
Total		9702	7573	2129

MEDLINE

#	Searches	Results	Comment
1	Influenza, Human/	51178	
2	influenzavirus a/ or influenza a virus/ or influenza a virus, h1n1 subtype/ or influenza a virus, h3n2 subtype/ or influenzavirus b/ or influenza b virus/	38870	
3	(influenza* or flu).ti,ab,kf.	118045	
4	Influenza Vaccines/	23677	
5	or/1-4	125622	Influenza terms
6	intubation/ or intubation, intratracheal/ or critical care/ or Hospitalization/ or "length of stay"/ or patient admission/ or patient discharge/ or patient handoff/ or patient readmission/ or patient transfer/ or inpatients/	358684	
7	airway management/ or airway extubation/ or respiration, artificial/ or high-frequency ventilation/ or high-frequency jet ventilation/ or interactive ventilatory support/ or liquid ventilation/ or noninvasive ventilation/ or one-lung ventilation/ or positive-pressure respiration/ or continuous positive airway pressure/ or intermittent positive-pressure breathing/ or intermittent positive-pressure ventilation/ or ventilator weaning/ or tracheostomy/	90075	
8	ventilators, mechanical/ or ventilators, negative-pressure/	9401	
9	intensive care units/ or coronary care units/ or respiratory care units/	62810	

10	(icu or icus or (intensive adj2 care*)).ti,ab,kf.	154069	
11	mo.fs.	598976	
12	morbidity/ or incidence/ or prevalence/ or mortality/ or "cause of death"/ or fatal outcome/ or hospital mortality/ or mortality, premature/ or survival rate/	904733	
13	(morbidit* or mortalit* or death* or fatal* or survival*).ti,ab,kf.	2157692	
14	Frailty/	3812	
15	actuarial analysis/ or life tables/ or quality-adjusted life years/ or probability/ or odds ratio/ or proportional hazards models/ or risk/ or logistic models/ or protective factors/ or risk assessment/ or adverse outcome pathways/ or "healthcare failure mode and effect analysis"/ or risk factors/ or uncertainty/	1396943	
16	((hospitalization or hospitalisation or inpatient* or "in-patient*") adj2 rate*).ti,ab,kf.	24225	
17	(hospitalized or hospitalised).ti,ab,kf.	102919	
18	or/6-17]	4052794	Hospitalization or morbidity/mortality terms
19	5 and 18	30902	Base clinical set
20	limit 19 to "middle age (45 to 64 years)"	8091	
21	Middle Aged/ or ("middle-age*" or "middle age*" or "adults aged > 65 years" or "50-64 years").ti,ab,kf.	4495400	
22	20 or (19 and 21)	8157	Age group limit
23	limit 22 to yr="2010 -Current"	5304	Final Results – publication date limit
